# A Novel PCR-Free Ultrasensitive GQD-Based Label-Free Electrochemical DNA Sensor for Sensitive and Rapid Detection of *Francisella tularensis* †

**DOI:** 10.3390/mi15111308

**Published:** 2024-10-28

**Authors:** Sumeyra Savas, Melike Sarıçam

**Affiliations:** 1 Department of Clinical Microbiology, Medical School, Bandirma Onyedi Eylul University, Bandırma 10200, Türkiye; 2 CBRN Defense Technologies R&D Group, Materials and Process Technologies, The Scientific and Technological Research Council of Türkiye (TÜBİTAK), Marmara Research Center, Kocaeli 41470, Türkiye; melike.saricam@tubitak.gov.tr

**Keywords:** DNA biosensor, *Francisella tularensis*, graphene quantum dots, infection disease, label-free biosensor

## Abstract

Biological warfare agents are infectious microorganisms or toxins capable of harming or killing humans. *Francisella tularensis* is a potential bioterrorism agent that is highly infectious, even at very low doses. Biosensors for biological warfare agents are simple yet reliable point-of-care analytical tools. Developing highly sensitive, reliable, and cost-effective label-free DNA biosensors poses significant challenges, particularly when utilizing traditional techniques such as fluorescence, electrochemical methods, and others. These challenges arise primarily due to the need for labeling, enzymes, or complex modifications, which can complicate the design and implementation of biosensors. In this study, we fabricated Graphene Quantum dot (GQD)-functionalized biosensors for highly sensitive label-free DNA detection. GQDs were immobilized on the surface of screen-printed gold electrodes via mercaptoacetic acid with a thiol group. The single-stranded DNA (ssDNA) probe was also immobilized on GQDs through strong π−π interactions. The ssDNA probe can hybridize with the ssDNA target and form double-stranded DNA, leading to a decrease in the effect of GQD but a positive shift associated with the increase in DNA concentration. The specificity of the developed system was observed with different microorganism target DNAs and up to three-base mismatches in the target DNA, effectively distinguishing the target DNA. The response time for the target DNA molecule is approximately 1010 s (17 min). Experimental steps were monitored using UV/Vis spectroscopy, Atomic Force Microscopy (AFM), and electrochemical techniques to confirm the successful fabrication of the biosensor. The detection limit can reach 0.1 nM, which is two–five orders of magnitude lower than previously reported methods. The biosensor also exhibits a good linear range from 10^5^ to 0.01 nM and has good specificity. The biosensor’s detection limit (LOD) was evaluated as 0.1 nM from the standard calibration curve, with a correlation coefficient of R^2^ = 0.9712, showing a good linear range and specificity. Here, we demonstrate a cost-effective, GQD-based SPGE/*F. tularensis* DNA test suitable for portable electrochemical devices. This application provides good perspectives for point-of-care portable electrochemical devices that integrate sample processing and detection into a single cartridge without requiring a PCR before detection. Based on these results, it can be concluded that this is the first enzyme-free electrochemical DNA biosensor developed for the rapid and sensitive detection of *F. tularensis*, leveraging the nanoenzyme and catalytic properties of GQDs.

## 1. Introduction

*Francisella tularensis* (*F. tularensis*), the causative agent of tularemia, is a small, facultative, and nonmotile Gram-negative coccobacillus [1]. That agent is highly infective, and only few microbes inhaled from the surrounding air are needed to start the disease. Also, bites by infected vectors, contact with infected animals, contaminated dust and aerosols, contaminated surfaces, or ingestion of contaminated food and water can all contribute to the spread of tularemia. It has the capability of causing high-consequence events for countries’ economies and creating threats to public health and safety. Because of that, the CDC (the Centers for Disease Control and Prevention) has cited *F. tularensis* as a potential biological weapon in the category A [2].

Several techniques have been used for the detection of *F. tularensis*, including cultivation methods and polymerase chain reaction (PCR)-based tests and biosensors. Although the culture method is the most widely used method, it has some major limitations, such as being fastidious, slow growing (up to 10 days), difficult to identify, and requiring a long time for analysis [3]. The PCR method is better than cultural methods; however, the method has various limitations, such as the requirement of expensive reagents, sophisticated instrumentation, qualified staff, and a time-consuming sample preparation process [4]. Biosensors offer strong alternatives to these techniques for the rapid and sensitive quantification of biological agents. Electrochemical DNA biosensors, in particular, have attracted great interest from the research community as an ideal disjunctive technique for pathogen detection due to their simple working principles, high sensitivity and low cost, miniaturization feature, compatibility with micro fabrication systems, and real-sample detection [5,6]. Among electrochemical sensors, the most commonly used are amperometric sensors, voltammetric sensors, and differential pulse voltammetric (DPV) sensors. Among these, voltammetric sensors such as linear sweep voltammetry or cyclic voltammetry require more complex instrumentation compared to amperometric sensors. Additionally, interpreting voltammograms can be challenging, especially in mixtures. Amperometric and DPV sensors have the highest sensitivity and selectivity, but DPV instrumentation and analysis are more complex compared to amperometric sensors. Furthermore, in amperometric sensors, it is possible that the current response is directly related to the analyte concentration. Additionally, due to their easier application to portable devices, amperometric sensors are more commonly preferred in biological studies [7,8,9]. Considering recent published studies, electrochemical DNA biosensors have the capability of detecting mutation, DNA damage, and even specific gene sequences of tough bacteria [6,10,11,12,13]. With this, a DNA biosensor allows for detection of analytes in body fluids directly on the basis of the nucleic acid hybridization principal without any requirement of enzyme-based target amplification, as with a PCR [6].

Gene-specific DNA-based biosensors usually consist of single-stranded DNA (ssDNA) samples known as capture DNA probes [14]. The strand is hybridized on the electrode surface after the capture DNA immobilization process, and target pathogen specific DNA can be detected without any amplification method. In electrochemical biosensors, pencil graphite electrodes (PGEs), screen-printed gold electrodes (SPGEs), and carbon electrodes (CEs) are used as transducers [15,16,17]. This type of biosensor converts the biological reaction between the bioreceptor and a specific target into a readable electrical signal which is proportional to the specific target concentration [18].

In biosensor applications, nanomaterials play a very important role in the development of various biosensors for disease detection due to their superior properties, such as high specificity, sensitivity, and reproducibility [5,6]. Among them, Graphene Quantum dots (GQDs) have attracted great interest because of their biocompatibility, large surface area, and suitability for chemical modification, as well as their ability to act like nanoenzymes. An enzyme is generally needed for the detection of the analyte by an electrochemical biosensor, and the Horseradish peroxidase (HRP) enzyme is the most commonly used enzyme in the studies [19]. For the determination of the analyte, generally, the secondary receptor is a requirement to be labeled with the enzyme, which results in longer and higher cost detection regarding the analyte. GQDs exhibit peroxidase-like catalytic activity and are capable of catalyzing hydrogen peroxide. GQDs also act as a nanoenzyme and eliminate the need for the use of HRP-labeled secondary structures. Through these advantages, GQD-based electrochemical biosensors have been chosen to determine biological agents rather than enzyme-based biosensors in order to enhance simple, fast, low-priced and sensitive detection techniques [20,21].

Nanoenzymes are generally categorized into two groups based on the reactions of the enzymes they mimic. The first group includes nanoenzymes that mimic oxidoreductases, such as glutathione oxidase and peroxidase, while the second group consists of nanoenzymes that imitate hydrolases like esterases, phosphatases, and proteases [22,23]. However, the latest technology has added a new category to these. These nanoenzymes, identified as artificial enzymes, are classified into four groups based on the most commonly used material type: metallic nanoenzymes such as gold and silver, metal oxide nanoenzymes, carbon-related nanoenzymes like functionalized graphene or Graphene Quantum dots, and other 2D nanomaterials [24,25]. Their tunability can be simply adjusted by changing elements, sizes, surface properties, and morphology of the dots. The exceptional chemical and physical properties of nanomaterials allow for infinite functionalization and modification, which easily produces nanozyme materials. Many studies have shown that GQDs possess peroxidase (POD)-like catalytic activities involving the oxidation of an electron-donating substrate and the simultaneous reduction in hydrogen peroxide (H_2_O_2_) [26,27,28]. Horseradish peroxidase (HRP) is the most commonly used peroxidase in biosensor applications, requiring the labeling of a secondary receptor for target identification. HRP-based systems acting as nanoenzymes can be replaced with GQDs, allowing for label-free detection of analytes. Guo and others mounted GQDs onto a gold electrode, where the GQDs still exhibited strong peroxidase-like properties even after being mounted on gold. The prepared GQDs-Au electrode showed good electrochemical catalytic properties against H_2_O_2_ decomposition. Electrochemical measurements demonstrated that the GQDs-Au electrode exhibited a rapid amperometry response, a wide linear range, and a low detection limit for H_2_O_2_ [29]. Recently, Yang and colleagues reported a label-free electrochemical immunosensor where GQDs effectively reduced H_2_O_2_, allowing for the sensitive detection of carcinoembryonic antigen (CEA) in human serum [30]. Studies have also achieved analyte quantification based on the reduction in H_2_O_2_ by GQDs. While the bare gold electrode surface could not reduce H_2_O_2_ on its own, our previous study proved that the GQD-modified gold electrode significantly reduced H_2_O_2_, producing gradually increasing signals in the μA range according to the increasing GQD concentration used. The enhanced electrocatalytic property of the GQD-modified electrode can be attributed to the close electronic interactions between Au and GQDs, which improve electron transfer [20,31].

In this study, a novel, fast, enzyme-free and sensitive electrochemical DNA biosensor was fabricated to detect *F. tuleransis*. To achieve this goal, the activated GQDs were laminated on a screen-printed gold electrode prior, and then neutravidin (NA) was linked on GQD surfaces via carbodiimide chemistry in order to conjugate GQDs with custom-designed biotin-labeled single-stranded capture DNA (ssDNA). After functionalization of GQD surfaces with ssDNA, the free and active space of the electrode surface was covered and deactivated using bovine serum albumin (BSA). The denaturated target *F. tuleransis* DNA (ssDNA) in the sample was hybridized with ssDNA on GQDs without any amplification requirement. The hybridized double-stranded DNA was measured via amperometric and voltammetric methods thanks to the degree of the inhibited electron transfer of GQDs on the Au electrode. Although electrochemical biosensors utilizing GQD particles with nanoenzyme properties already exist in the literature, this study is the first to develop an enzyme-free electrochemical DNA biosensor for the rapid and sensitive detection of *F. tularensis*.

## 2. Materials and Methods

### 2.1. Materials and Reagents

N-hydroxysuccinimide (NHS), ethanolamine, molecular grade ethanol, hydrogen peroxide, 11-Mercaptoundecanoic acid (MUDA), phosphate-buffered saline tablets (PBS, 0.01 M phosphate buffer, 0.0027 M potassium chloride and 0.137 M sodium chloride, pH 7.4). Graphene Quantum dots (<5 nm) were purchased from Sigma-Aldrich (Poole, UK). Neutravidin (NA) and 1-Ethyl-3-(3-dimethylaminopropyl)-carbodiimide (EDC) were sourced from Thermo Fisher Scientific (Loughborough, UK). Double-distilled ultra-pure water (18 MΩ cm^−1^) was produced by a Milli-Q water system (Millipore Corp., Tokyo, Japan). Synthetic oligonucleotides designed for the identification of the pathogenic bacterium *F. tularensis*, including the sequences of capture probe DNA (ssDNA) and complementary linear target DNA (ssDNA), were purchased from TIB Molbiol (Berlin, Germany). The oligonucleotide sequences (5′ to 3′) were determined by bioinformatic analysis and contain sequence motifs capable of hybridizing with capture DNA, as listed in Table 1. All voltametric and amperometric measurements were conducted using a Metrohm DropSens MicroStat 8000 Electrochemical Analyzer with the general purpose electrochemical software Dropview 1.4 (Dropsens, Asturias, Spain) and Metrohm DropSens screen-printed gold electrodes that are compatible with the device.

The detection of the *F. tularensis* tul4 gene was the primary focus. For alignment, sequences M32059, EF208970, EF208977, and EF208979 were used, with M32059 sequences being considered as the reference. Initially, 60 nucleotide contigs were generated to identify potential probes. Probes conserved in the *Francisellaceae* genome were eliminated, as they could indicate contamination. The remaining probes were screened against a set of non-*Francisellaceae* bacterial genomes available in GenBank. The clustering of the probes was carried out using the USEARCH v11 program (QIIME2). The final probe was synthesized by TIB Molbiol (Berlin, Germany), with the capture probe being biotin-labeled and amin-functionalized at the 5′ end.

### 2.2. Selection of Optimal GQDs Concentration for Bioassays and Preparation of SAM and GQDs Modified SPGE

First, the electrodes were washed with pure ethanol and double-distilled water, then thoroughly dried using a stream of nitrogen gas. Before creating a self-assembled monolayer (SAM) on the sensor chip, a thiol solution with a 2 mM concentration of mercaptoacetic acid (MUDA) in absolute ethanol was prepared for SAM deposition. The sensor chips were soaked in this ethanolic solution overnight, then washed with ethanol and Milli-Q water in sequence. Subsequently, the chips were thoroughly dried under a nitrogen gas stream, vacuum-sealed, and stored at +4 °C until needed [32]. To select the optimal GQD concentration for bioanalysis, the optical properties of GQDs and the possibility of saturation at decreasing concentrations were evaluated using an amperometric technique and UV/Vis spectroscopy, respectively [20]. Three different GQD suspensions in double-distilled ultra-pure water (50,000 ppm, 5000 ppm, and 500 ppm) were scanned from 300 nm to 700 nm using UV/Vis spectroscopy to determine the maximum absorbance value. Additionally, screen-printed gold electrodes were immersed in an ethanol solution overnight, followed by rinsing with ethanol and ultra-pure Milli-Q water before being dried individually [33]. Subsequently, GQD suspensions at decreasing concentrations were drop-cast and dried on the bare electrode surfaces. Amperometric measurements were then performed to determine the optimal GQD concentration for bioanalysis and establish the signal/concentration relationship. Each experiment was repeated three times for each concentration, and the optimum GQD concentration was determined using either optical or electrochemical methods.

### 2.3. Capture Probe ssDNA Immobilization and Characterization

**Figure 1:**
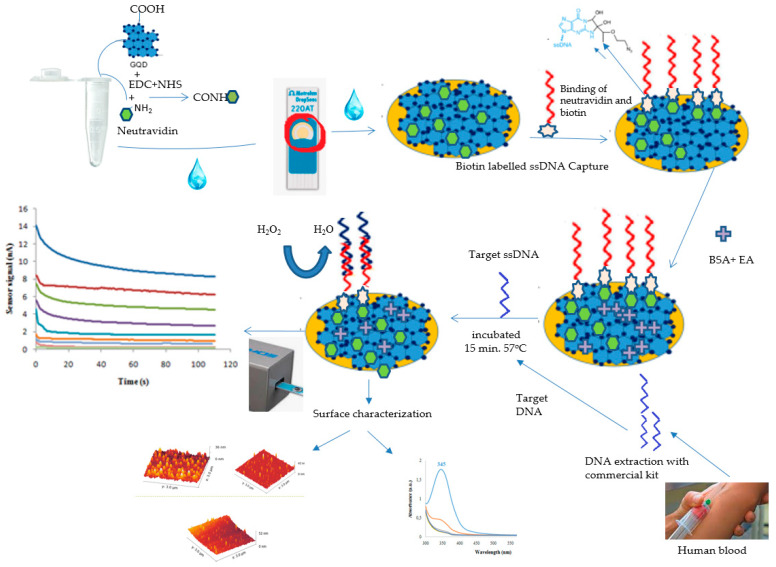
Principle of the GQD-based DNA sensor for *F. tularensis* detection.

Before preparing the nanohybrid, GQDs were activated and conjugated with NA using carbodiimide chemistry. A 5000 ppm GQDs solution was prepared by diluting with water. Initially, 50 μL of GQDs (0.5 M solution) were mixed with 50 μL of EDC (1.0 mg/mL solution) and 50 μL of NHS (1.0 mg/mL solution) in 250 μL of 1× PBS. This mixture was incubated at room temperature (RT) for 25 min. After incubation, 0.1 μg of NA was added, and the mixture was incubated for another 15 min at RT. To further enhance the conjugation efficiency of the amine-carboxyl coupling reaction, an additional 50 μL of EDC and 50 μL of NHS were added to the conjugate mixture, which was then stirred for 15 min at room temperature. The reaction mixture was then stirred for 8 h at 4 °C using a shaker. Following the EDC/NHS process, the modified SPE was sterilized using 70% ethyl alcohol and then allowed to dry inside the biological safety cabinet. After this, free GQDs were separated from the neutravidin-coated GQDs by centrifugation at 6000 rpm for 15 min. The supernatant was removed, and the neutravidin-conjugated GQDs conjugates were prepared [34,35]. After the all period, 125 µL of the resulting solution was placed onto the SPGE and left overnight in order for GQDs to laminate onto the sensor surface. The next day, the sensor surface was washed first with phosphate-buffered saline (PBS, pH 7.4) and then with ultrapure water to removed unbound EDC/NHS and dried. The optimized capture DNA concentration from our previous study was used in this work [33]. Capture DNA at a 10 µM concentration was sent to the sensor surface and incubated for 1 h [5,32,33]. Biotin-labeled custom-designed capture ssDNA was covalently attached on the surface. Subsequently, the immobilization process was performed, and each step included coating the surface with 100 µg/mL BSA prepared in PBS buffer and 1 M pH 8.5 ethanolamine for 4 min each to block areas without NA [33]. Each step of capture DNA immobilization was carried out at room temperature in a Petri dish to protect from environmental contamination and evaporation (Figure 1). During each step of capture probe immobilization, the sensor surface was carefully washed with PBS buffer (pH 7.4) to remove weakly adsorbed capture probes and other substances. Bare screen-printed gold, GQD-laminated, and capture DNA-immobilized sensor surfaces were characterized using an Atomic Force Microscope (AFM) (Naio, Nanosurf AG, Liestal, Switzerland). Commercial AFM probes (NCLR) provided by NanoWorld (NanoWorld AG, Liestal, Switzerland) were used for measurements with a spring constant of 23.191 N/m, resonant frequency of 170.481 Hz, scan size of 3.027 µm, and scan speed of 2 s. All AFM measurements were conducted at room temperature in intermittent air modes.

### 2.4. DNA Detection Assay

The detection assay of *F. tularensis* via a GQD-based electrochemical biosensor was based on the hybridization between the denaturated target ssDNA and the capture ssDNA-immobilized SPGE. The 5000 ppm GQDs, which were evaluated as optimal concentration for the assay, was immobilized on the SPGEs. ssDNA-captured DNA in the range of 0.01 nM–10^5^ nM was conjugated on the GQD-immobilized chips. The 10^5^ nM target ssDNA was sent on the chip surface and hybridized at 57 °C for 10, 15, 20, and 30 min intervals. As a result of the amperometric measurement results, the optimal hybridization time was determined. Additionally, the 3 × 3 µm^2^ areas of the GQD-immobilized, capture ssDNA-functionalized, and target ssDNA-hybridized SPGEs were characterized by AFM to visualize the 3D surface topography.

### 2.5. Selectivity, Specificity, and Stability of a GQD-Based Electrochemical DNA Biosensor

The electrochemical selectivity, specificity, and stability of the developed DNA biosensor are directly related to the differentiation of target ssDNA sequences (*F. tularensis*) from other bacterial genomic DNAs using amperometric measurements. *Salmonella* spp. and *Yersinia pestis* (*Y. pestis*) were chosen as model bacteria, and the target ssDNA sequences are given in Table 2. The target ssDNA concentration for all three biological agents was set at 10 ng/μL [36]. The amperometric current values were measured before and after the hybridization of the DNA biosensor with a target ssDNA of *F. tularensis*, *Salmonella* spp., and *Y. pestis*, and the selectivity, specificity, and stability of the DNA biosensor were evaluated. Additionally, results from hybridization with a 3-base mismatched complementary target, a 2-base mismatched complementary target, and a 1-base mismatched complementary target were compared with those of non-complementary DNA and target DNA. The specificity of the untagged form of the target DNA was confirmed via PCR testing. For this, the forward primer, GCT GTA TCA TCA TTT AAT AAA CTG CTG, and the reverse primer, TTG GGA AGC TTG TAT CAT GGC ACT, pair were used, and the tul4 gene of 420 bp in size was detected by a gel image traditional polymerase chain reaction.

## 3. Results and Discussion

### 3.1. Optimization of GQDs Concentration for Bioassays

**Figure 2:**
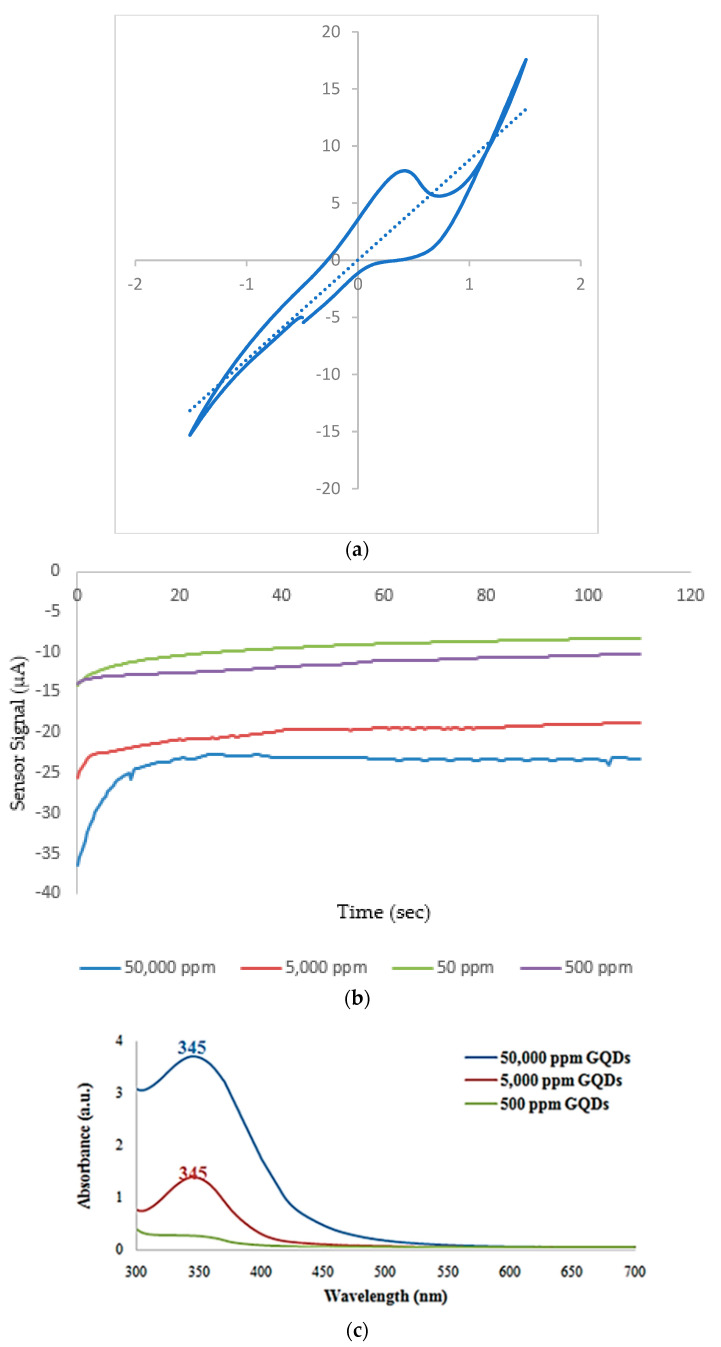
(**a**) Cyclic voltammetry measurement to determine the most ideal amperometric measurement. (**b**) Real time measurement curves obtained with four different GQDs concentrations. (**c**) Optical properties of GQDs at decreasing concentrations.

The saturation profile and optical properties of GQDs in terms of concentration were investigated by amperometry and UV/Vis spectroscopy, respectively. The catalytic surface area and high electrical conductivity created by the electronic interaction between the GQDs with a diameter of less than 0.005 μm and the screen-printed gold electrode (SPGE) surface created a highly active electrocatalyst effect for the reduction in H_2_O_2_ [37,38]. In the study, first the appropriate GQD concentration was determined. Cyclic voltammetry measurements were made with a concentration of 50,000 ppm GQDs. As a result of the cyclic voltammetry measurement taken in the −2.0 +2.0 V range, the correct measurement value was determined for the amperometry measurement, with the peak occurring above −0.2 V. The cyclic voltammetry results for stock GQDs (50,000 ppm) were given in Figure 2a, and the electrochemical signal was carried out at −0.2 V with constant potential. The amperometry measurement of GQDs at decreasing concentrations was shown in Figure 2b, and it was seen that a biosensor-signaling value difference of between 50,000 ppm and 5000 ppm was at the approximate level of 2 μA. Based on the results, and our previous study experience, the optimum concentration of GQDs was determined as 5000 ppm to develop a cost-effective process since the signaling between biosensors can be negligible [20]. Figure 2c demonstrates the optical properties of the GQDs used in this study. The concentration dependent UV/Vis spectra of GQDs indicates a peak at 345 nm which is related to the electronic transition from n−π* assigned to the C=N and C=O of the GQDs. While the typical absorption peak on the spectra of GQDs at 50,000 and 5000 ppm was sharp, a peak of 500 ppm is not remarkable. This consequence also assigns the optimal concentration of GQDs as 5000 rpm for DNA biosensors.

### 3.2. Label-Free DNA Biosensor for F. tularensis Detection

**Figure 3:**
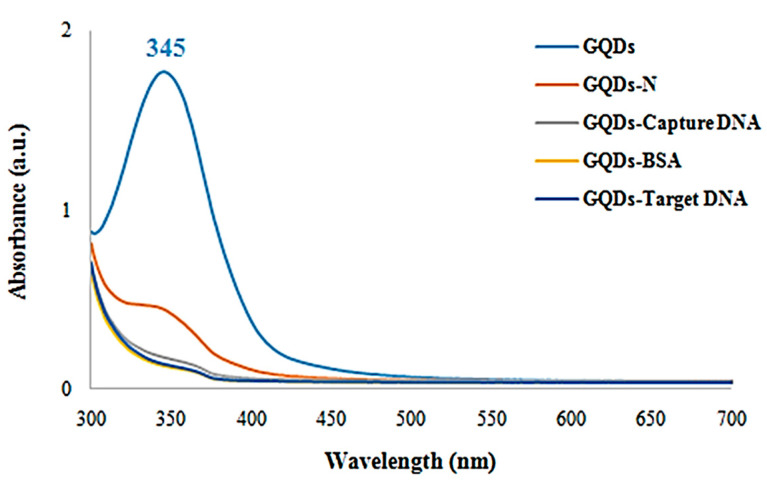
Comparative UV/Vis spectra of GQDs, GQDs-N, GQDs-Capture DNA, GQDs-BSA and GQDs-Target DNA.

In this study, we first determined the suitable amount of GQDs for use in biosensor development based on the signal–concentration relationship. The GQDs resulted in sensor signals in the µA range on the bare SPGE surfaces. The GQDs were conjugated with capture ssDNA through amide bond formation by linking the –NH_2_ group of NA and the –COOH group on the GQD surfaces. The NA-linked GQDs (GQDs-N) reacted with the biotin-labeled capture ssDNA. The biotin–neutravidin binding mechanism is a highly specific and strong non-covalent interaction that is widely used in biotechnology and biosensor applications. Neutravidin is a deglycosylated form of avidin, a protein derived from *Streptavidin*, but it is modified to reduce non-specific binding while maintaining a strong affinity for biotin. The binding site of neutravidin is a deep pocket that perfectly fits the biotin molecule. This structural compatibility contributes to the high specificity of the interaction. The conjugate (GQDs-Capture DNA) was blocked with BSA as a final detection product (GQDs-BSA) in order to prevent non-specific binding. The whole conjugation and target DNA detection reactions were confirmed by UV/Vis spectroscopy via investigating the absorption properties of GQDs, as seen in Figure 3. The characteristic peak of the bare GQDs was seen at 345 nm. On the other hand, the spectrum was dramatically altered after functionalization with NA, during which the absorption peak was redshifted and became weaker. With the addition of capture ssDNA, the absorbance around the typical peak disappeared. Furthermore, no significant absorbance was seen on the spectra of GQDs-BSA and GQDs-Target DNA (target DNA added the BSA-blocked GQDsconjugate). This result shows that the GQD surfaces on SPGE were successfully conjugated with capture ssDNA and that the conjugation process was followed via UV/Vis spectroscopy.

**Figure 4:**
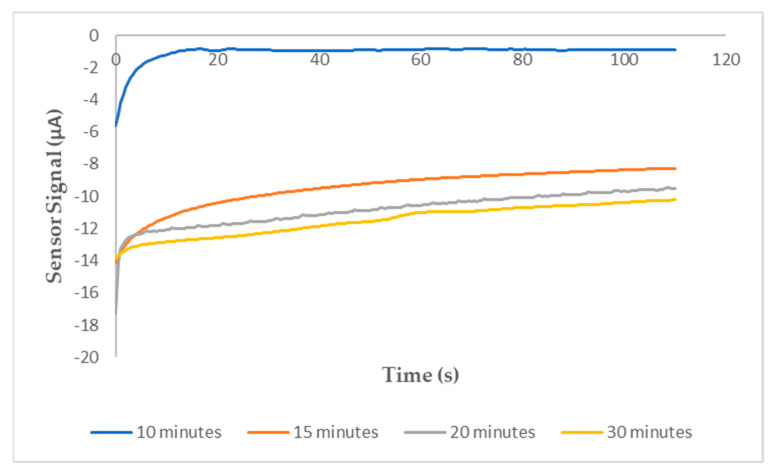
Optimization of the ideal hybridization time between capture DNA (ssDNA) and 10^5^ nM target DNA (gDNA) on the sensor surface.

The hybridization time of capture ssDNA on SPGE and target ssDNA was optimized to achieve the high performance of the biosensor. For this reason, the hybridization of 10^5^ nM target ssDNA and capture ssDNA on SPGE for 10, 15, 20 and 30 min was investigated in triplicate sets; the comparative sensor signal caused by the hybridization time is seen in Figure 4. It was observed that no significant hybridization occurred in the 10th minute and that the capture and target ssDNA were hybridized at around 15 min. Moreover, the sensor signals of hybridization at the 20th and 30th minutes were similar. Considering all these results, it was decided that the optimal hybridization time for capture ssDNA on SPGE and target *F. tularensis* ssDNA was 15 min.

**Figure 5:**
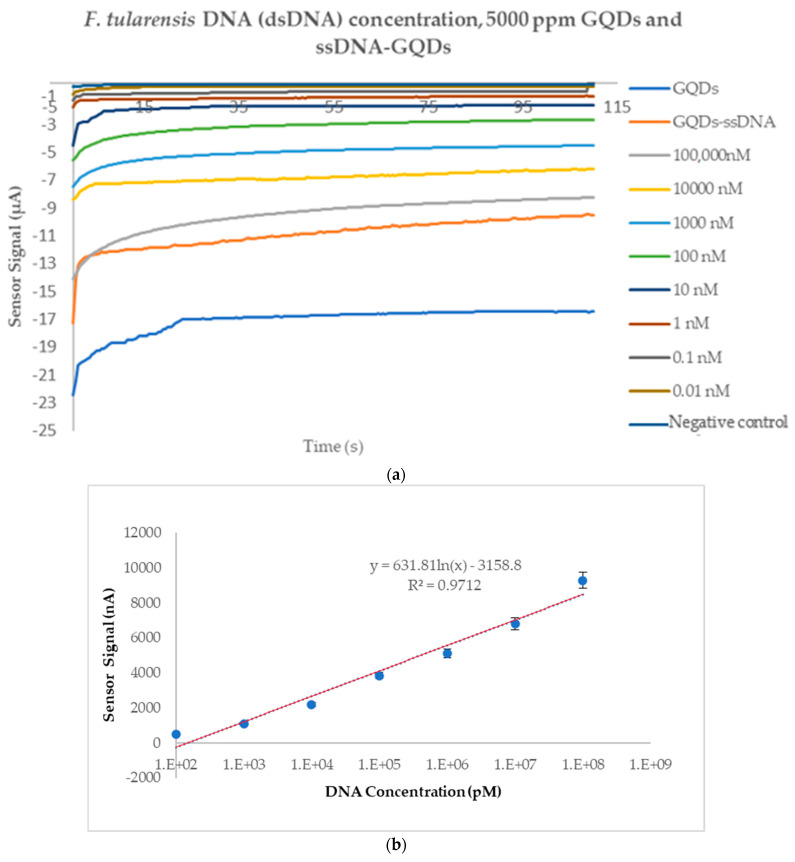

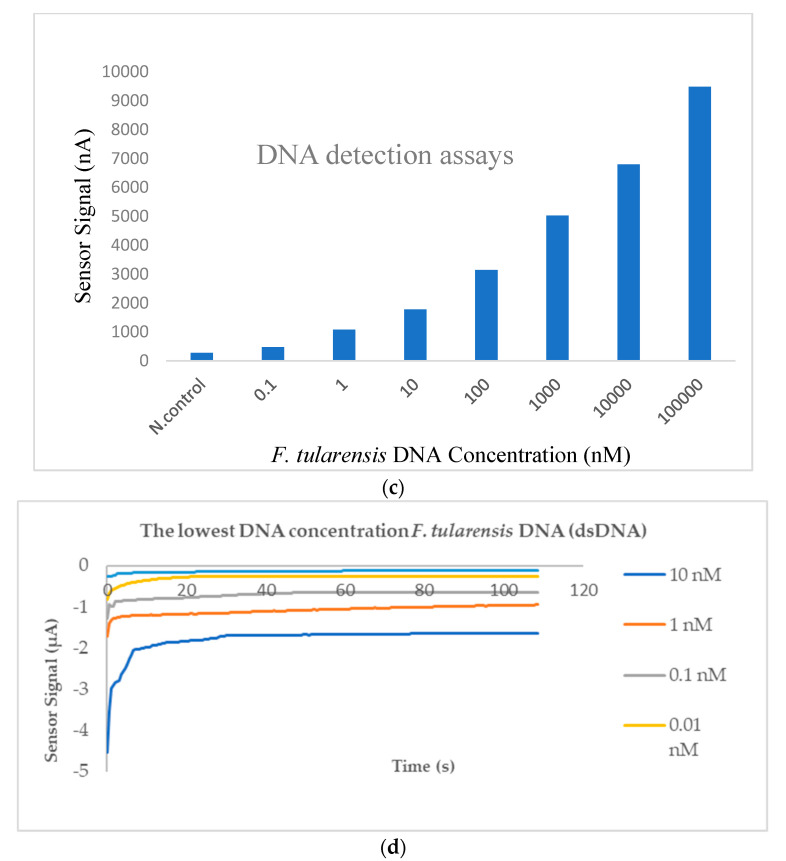
(**a**) The amperometric measurement curves obtained for eight concentrations of *F. tularensis* DNA (dsDNA) concentration using 5000 ppm GQDs, 5000 ppm GQDs, and GQDs-ssDNA attachment. (**b**) The linear calibration curve of DNA assays (10^5^ nM–0.1 nM) with a correlation coefficient of 0.9712. (**c**) Sensogram of eight concentrations of *F. tularensis* DNA (dsDNA). (**d**) The amperometric measurement curves obtained only between 10 nM–0.01 nM.

For the first time, we investigated the potential of a GQD-based enzyme-free DNA biosensor for *F. tularensis* without using a PCR. The detection of target *F. tularensis* DNA was examined in the range of 10^5^ nM–0.1 nM using a DNA surface probe captured by a neutravidin-immobilized layer on the sensor chip. Eight different concentrations (10^5^ nM–0.1 nM) of the *F. tularensis* DNA target were prepared in deionized water. GQDs produced sensor signals in the µA/nA range on bare screen-printed gold electrode surfaces. However, when we measured the highest DNA concentration (10^5^ nM), the sensor signal was observed to be around −8 µA (Figure 5). The lowest DNA concentration, 0.1 nM, generated an electrochemical signal of −650 nA. In our previous study, we developed an electrochemical measurement-based immunoassay and DNA sensor labeled with a gold nanomaterial using a HRP enzyme-labeled polyclonal antibody (with TMB as the substrate) which was capable of detecting *Salmonella* spp. Subsequently, to observe the nanoenzyme effect of GQDs, as mentioned in the literature, we developed an immunoassay using GQDs in place of the HRP-TMB complex for the detection of *Yersinia enterocolitica* in milk. When we compared the studies we conducted, the excellent electrical conductivity and increased surface area of the GQD sensor led to improved detection. Comparing this result with the HRP enzyme-labeled AuNP-amplified immunoassay and DNA biosensor mentioned above, we observed that the detection limit decreased from 20 nM to 0.01 nM and that the sensitivity increased by 2–5 times [20,32]. Additionally, the current biosensor strategy provides easy-to-apply sensor arrays as it does not require an extra step for the conjugation of nanomaterials with the detection probe [20]. In this way, the use of GQDs significantly reduces detection time and lowers costs. Figure 5a shows the electrochemical measurement values for all concentrations with bare GQDs and a GQDs-ssDNA complex. As the ssDNA target concentration increases, a positive shift in the electrochemical signal values on the transfer curves was observed, as seen in many electrochemical studies [5,39,40,41,42,43].

After binding with ssDNA-QDs in the presence of single-stranded bacterial DNA (synthetic target DNA of *F. tularensis*) obtained after denaturation, the ssDNA-QDs initially bind to the target DNA. In this case, the nucleobases in the ssDNA complex bound to the QDs are hidden within the helical structure, leaving only negatively charged phosphate groups in the environment. Compared to bare GQDs, after the signal drop observed in the ssDNA-GQD complex, the ssDNA (capture DNA) is released from the QD following the hybridization of the target DNA and ssDNA (capture DNA), and an increase in the electrochemical sensor signal is observed as the target DNA concentration increases. Moreover, another reason for this, according to the literature, is conductivity. The goal is for the target to cover the surface, making it easier for electrons to reach the surface with a higher current. Studies have shown that dsDNA, which has a more stable structure, is more conductive than ssDNA. Additionally, the ratio of G-C base sequences to A-C base sequences used in bioinformatic analyses significantly affects conductivity. The structure, composition, sequence, and length of DNA greatly influences conductivity [44,45]. In our study, due to the conductive structure of the ssDNA used, conductivity increased, and the stable structure of dsDNA further increased conductivity, leading to an increase in concentration-dependent electrochemical measurements. In a study, the effect of five different DNA sequences on DNA conductivity was investigated. It was observed that there was a transition from a semiconductor region to a high-conductivity region during the transition from ssDNA to dsDNA (after hybridization). The higher conductivity observed in dsDNA samples compared to ssDNA was attributed to the greater stability of the dsDNA molecules. In dsDNA, each base is bound to its complementary base via hydrogen bonds, whereas single-stranded bases can form hydrogen bonds with other molecules, such as water [44]. The hydrogen bonds between complementary bases in dsDNA make it stiffer and more stable than ssDNA [45,46]. Additionally, the double helical structure is more suitable for the movement of charge carriers during hopping or tunneling, as explored and explained in studies on double-stranded structures [47,48]. Moreover, the lower base stacking in ssDNA compared to dsDNA may reduce the overlap potential of π-electrons, affecting conductivity. Furthermore, dsDNA has greater periodicity in its structure, which is more favorable for hopping states that better preserve polarization energy [45]. In general, the number of nitrogenous bases in dsDNA is twice that of ssDNA of the same length, which can affect the number and movement of charge carriers and thus influence conductivity. In this study, the genomes of *F. tularensis* found in the NCBI system were systematically aligned and a sequence was generated as a result of a detailed computational analysis. In studies referenced with the same DNA sequence, it is possible for the results to be similar, depending on the DNA sequence and synthesis, as well as the formation of an inverse curve.

Figure 5b shows the amperometric value difference in the DNA biosensor as a function of the logarithmic concentration of the target ssDNA (Log DNA). A good linear relationship with a value of R^2^ = 0.9712 was obtained with the logarithmic concentrations of the DNA targets. The detection of *F. tularensis* DNA was investigated in a concentration range of 10^5^ nM–0.1 nM using the immobilized DNA capture probe on the sensor surface, and good reproducibility was shown over a wide investigation range. Figure 5d is provided solely to show the difference between low concentrations. As shown in Figure 5d, the difference between the lowest DNA concentration (0.01 nM) and the negative control was observed. However, since the 0.01 nM concentration was close to the negative control (negative control: −169 nA; 0.1 nM: −650 nA; 0.01 nM: −193 nA), the lowest measurable concentration was accepted as 0.1 nM.

When the target DNA was added, the hybridized dsDNA molecules detached from the GQDs, and the shift in the transfer curves only along the gate voltage axis without a shift in the channel current axis indicated that the change in the channel current was due only to the horizontal shift of the transfer curve. Therefore, the amperometric current change is related only to the target DNA concentration. The concentration of the target DNA can be calculated by measuring the amperometric current. Real-time measurement can be achieved. Electrochemical current measurement is highly sensitive and allows for real-time measurement [49]. As a result, the sensor’s electrochemical signal response to target DNA was investigated.

Figure 5a shows the hybridization process of the target DNA with the probe ssDNA on GQDs and the detachment of the hybridized dsDNA from the GQD surfaces. When the target DNA was added to the PBS solution, the sensor signal initially increased and gradually stabilized. These changes can be explained in two stages: first, the target DNA hybridizes with the probe ssDNA, which is similar to applying a negative voltage and initially increases the signal. Then, finally, when all target DNA hybridization is complete, the amperometric measurement stabilizes.

Thus, real-time signal changes allow for monitoring of DNA hybridization and detachment from GQD surfaces, enabling real-time DNA detection. Figure 4 shows the hybridization time of the DNA target, indicating an equilibrium time of 1010 s when the target DNA was added to the ssDNA. Minimal changes after 15 min suggest the DNA biosensor’s capability to rapidly detect ultra-low DNA concentrations.

**Figure 6:**
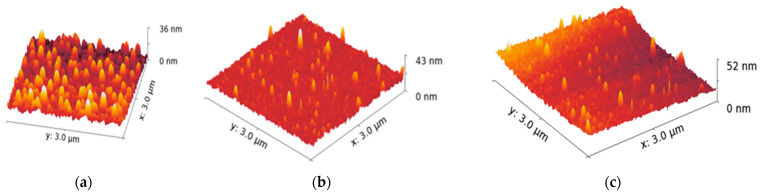
AFM 3D topography images of (**a**) GQD-laminated surface, (**b**) Capture DNA immobilized and (**c**) After hybridizationTarget DNA binding.

A GQD-laminated, capture ssDNA was immobilized, and, after hybridization with *F. tularensis* target ssDNA, the sensor surfaces were characterized by using AFM. The 3D surface topography images were analyzed in the scanning area of 3 × 3 µm^2^, as seen in Figure 6. The heights of GQDs on SPGE, capture ssDNA-immobilized SPGE, and *F. tularensis* target ssDNA-hybridized SPGE were measured as 36, 43 and 52 nm, respectively. The gradual increment in the surface height shows the t successful surface functionalization of GQDs with capture ssDNA and hybridization of *F. tularensis* target ssDNA was monitored with AFM [50].

By measuring the genetic material of bacteria, DNA biosensors represent a powerful alternative to antibody biosensors for the diagnosis of pathogenic diseases, especially since they eliminate the possibility of non-specific binding. In the past experience of the team, the detection limit for *Salmonella* spp. genomic DNA, DNA with a specific DNA biosensor, was 20 nM. In this study, this concentration decreased more as 1 × 10^−4^ μM *F. tularensis* target ssDNA thanks to the nanoenzyme properties of GQDs. In other words, the use of GQD nanoparticles in the DNA biosensor development offers superior sensitivity that plays an important role while using direct genomic DNA for detection of pathogens. Another advantage of this study is that no pretreatment process like PCR, which prolongs the measurement time and increases the cost, is required. As a conclusion, this novel developed label-free DNA biosensor can open a new door to the sensitive detection of other biological warfare agents.

In the literature, it is seen that various nanomaterials are used while developing DNA-based electrochemical sensors. Gold nanomaterials as well as electrochemical biosensors, nanomaterials used in the formation of DNA biosensors are given in Table 3 for comparison purposes. However, as can be seen here, the measurement limits of electrochemical DNA biosensors developed using GQDs are 47% lower than the others. This shows that GQDs make a great contribution to the sensitivity of electrochemical DNA biosensors. The results of the study conducted by Ahao et al. on HBV-DNA also support our work and show the effect of carbon dots, GQDs, and similar nanomaterials on DNA biosensors.

There are various studies using DNA biosensors for the detection of pathogens. Ariffin et al. introduced an electrochemical DNA biosensor for *Escherichia coli* (*E. coli*) detection [53]. They used Au nanoparticles and determined the lowest detection limit as 1.95 × 10^−21^ M. Ilkhani et al. have fabricated a DNA biosensor for Ebola virus detection, developed a DNA biosensor using the enzyme, and determined the detection limit as 47 × 10^−4^ μM [5]. In addition, Ali et al. fabricated an enzymatic DNA biosensor using AuNPs for detection of *Vibrio cholerae*, and a limit of detection (LOD) value was calculated as 7.41 × 10^−30^ molL^−1^ [54]. Until the present study, traditional methods and PCR methods have been used in studies carried out for the detection of the relevant pathogen. However, these methods are time-consuming and require laboratory conditions. Moreover, at least 0.5–1.0 µM or 200 ng of bacterial DNA is needed for PCR initiation [55]. Nevertheless, *F. tularensis* is a potential bioterrorism threat due to its high virulence and low infective dose. A DNA biosensor developed specifically for *F. tularensis* has not yet been found in the literature, and this study has the distinction of being the first to investigate this concept. Tularemia is the most commonly reported laboratory-acquired bacterial infection. For this reason, it is very important to have a user-friendly, fast, sensitive, inexpensive system against possible threats.

**Figure 7:**
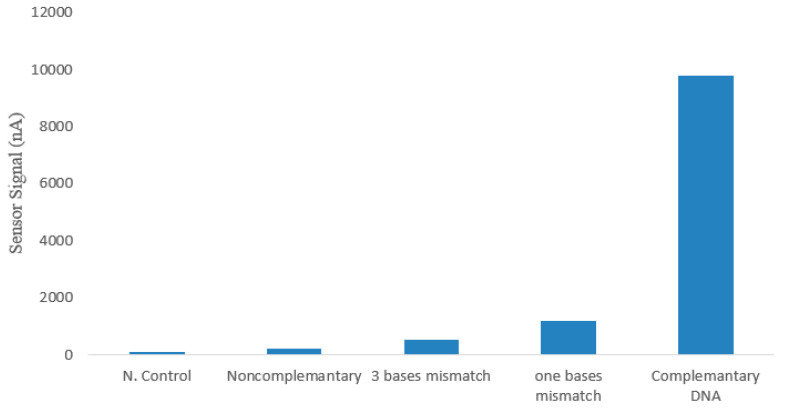
Current responses for the detection of non-complementary DNA targets (*Salmonella* spp. target *Y. pestis* target), three-base mismatched DNA targets, one-base mismatched DNA targets, and complementary DNA targets.

**Figure 8:**
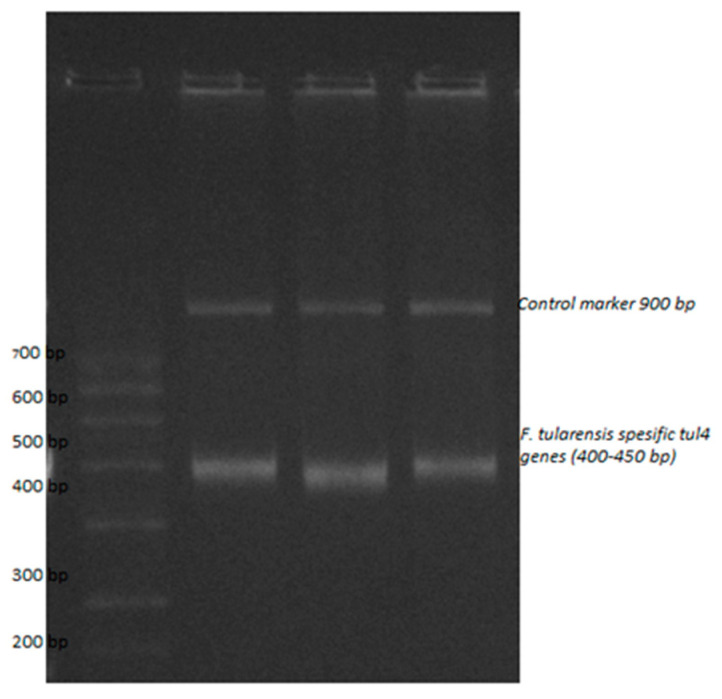
Gel electrophoresis experiments to verify the applicability of the DNA chains designed in the article (the forward primer: GCT GTA TCA TCA TTT AAT AAA CTG CTG and reverse primer: TTG GGA AGC TTG TAT CAT GGC ACT pair was used and a tul4 gene of 428 bp size was detected).

Specificity is also a very important parameter in evaluating the performance of a biosensor. The electrochemical signal values observed after the hybridization of mismatched ssDNA with three bases altered, single-base mismatched ssDNA, and complementary ssDNA were compared (Figure 7). It was found that, compared to the complete complementary DNA targets, the signal was nine times lower for the mismatched ssDNA due to the unstable double helix structure after hybridization. This is evidence that the biosensor has good specificity to the target DNA. After hybridization with two separate non-complementary ssDNA sequences, the obtained electrochemical signal was exactly the same as the negative control. This indicates that the DNA capture and complementary target DNA are specific to *F. tularensis* and that there is no possibility of cross-reaction. In Table 4, mismatched complementary DNA target sequences are provided. Specificity tests are consistent with the DNA biosensor study optimized by Deng et al., which also indicated no risk of cross-reaction [56]. Also, the study was supported by gel electrophoresis experiments to verify the applicability of the DNA chains designed in the article and is presented in Figure 8. The forward primer: GCT GTA TCA TCA TTT AAT AAA CTG CTG and reverse primer: TTG GGA AGC TTG TAT CAT GGC ACT pair was used and a tul4 gene of 428 bp size was detected (Table 4).

To assess reproducibility, accuracy, and stability, three separate measurements were taken for each concentration on three different chips. At the end of the experiment, each concentration was measured electrochemically three times. Reproducibility was calculated based on the coefficient of variation (CV). This value was determined using the following formula: when the standard deviation and average of all three measurements were calculated and compared, the CV% was found to be 3. A CV% value below 10 indicates that the experiment is reproducible [57].

**Figure 9:**
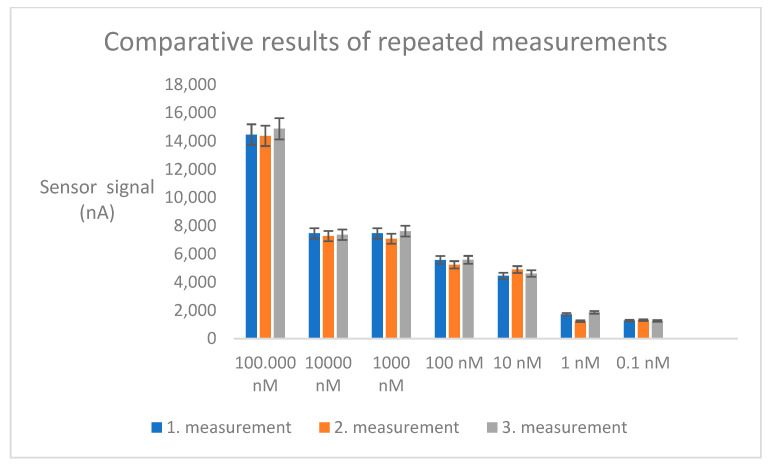
Comparison of the similarity ratios for the same concentrations measured in three different measurements.

The SSGE chips immobilized with ssDNA were individually opened at 1–8-week intervals to measure the target DNA at its maximum concentration (100 µM). As shown in Figure 9, the coefficient of variation between the electrochemical measurement results was calculated as 4 using the formula below. This result indicates that the sensitivity of the immobilized chips remained stable over an 8-week period. Additionally, Figure 9 shows a comparison of the similarity ratios for the same concentrations measured in three different measurements.

Coefficient of variation (CV) = Standart deviation (SD)/average of detection value × 100%f.

## 4. Conclusions

In this work, a novel label-free GQDs-based DNA biosensor was developed using an electrochemical transducer. The potential to measure the denaturated *F. tuleransis* genomic ssDNA at 10^5^ nM concentration without pretreatment for DNA analysis has been successfully demonstrated for the first time. The whole process of detecting the biological agent via this novel DNA biosensor takes 1010 s (17 min) maximum. It provided the highest selectivity and sensitivity among the methods used in the detection of *F. tuleransis*. The rapid, specific, label-free, and inexpensive biosensing system using the developed GQDs can be integrated into all electrochemical measuring devices and can be used in the field by making it portable.

## Figures and Tables

**Table 1:** Oligonucleotides sequences in the present.

DNA Primer	Sequence
Capture Probe (ssDNA) Complementary target (ssGDNA)	5′-TTCTCAGCATACTTAGTAATTGG 5′TCTAAGTGCCATGATACAAGCTTCCCAATTACTAAGTAT

**Table 2:** Non-complemanter DNA target with *F. tularensis*.

DNA Primer	Sequence
*Salmonella* spp. target *Y. pestis* target	5′-TTCTCAGCATACTTAGTAATTGG 5′TCTAAGTGCCATGATACAAGCTTCCCAATTACTAAGTAT

**Table 3:** Nanomaterials based biosensor performance for the detection of DNA.

Nanomaterials	Detection Methods	Target	Limit of Detection	References
GQDs	Electrochemical biosensor	HBV-DNA	1 nM	[6]
Neutravidin modified	Electrochemical biosensor	HBV-DNA	2350 nM	[51]
AuNPs	Electrochemical biosensor	ssDNA-AuNPs	13 nM	[52]
Enzyme-amplified	Electrochemical biosensor	Ebola Virus	4.7 nM	[5]
Carbon nanomaterials	Electrochemical biosensor	HBV-DNA	15 nM	[8]
GQDs	Electrochemical biosensor	*F. tularensis*	0.1 nM	This study

**Table 4:** Mismatched complementary DNA target sequences.

DNA Primer	Sequence
Complementary target (ssGDNA); Three-base mismatched complementary target; Two-base mismatched complementary target; One-base mismatched complementary target.	5′TCTAAGTGCCATGATACAAGCTTCCCAATTACTAAGTAT 5′TCTAAGTGCCATcATACAAGCTTgCCAATTACTtAGTAT 5′TCTAAGTGCCAacATACAAGCTTCCCAATTACTAAGTAT 5′TCTcGGTGCCATGATACAAGCTTCCCAATTACTAAGTAT

## Data Availability

The original contributions presented in the study are included in the article, further inquiries can be directed to the corresponding author.
